# Bridging the gap in AI integration: enhancing clinician education and establishing pharmaceutical-level regulation for ethical healthcare

**DOI:** 10.3389/fmed.2024.1514741

**Published:** 2024-12-19

**Authors:** Alessandro Perrella, Francesca F. Bernardi, Massimo Bisogno, Ugo Trama

**Affiliations:** ^1^I UOC Emergin Infectious Disease and High Countagiousness, AORN Ospedali dei Colli—P.O. D. Cotugno, Naples, Italy; ^2^Coordination of the Regional Health System, General Directorate for Health Protection, Naples, Italy; ^3^Department of Experimental Medicine, University of Campania Luigi Vanvitelli, Naples, Italy; ^4^Regional Special Office for Digital Transformation, Campania Region, Naples, Italy

**Keywords:** regulatory drug development, European Medicines Agency (EMA), artificial intelligence, antimicrobial stewardship (AMS), ethic and development, ethic, medical AI governance, pharmaceutical validation

## 1 Introduction

Recently the role of AI in healthcare has been deeply studied and discussed in scientific literature. Promising applications of artificial intelligence and machine learning (AI/ML) are revolutionizing both clinical and administrative domains, with significant advancements demonstrated in drug discovery, precise analysis and interpretation of radiological images, early and accurate sepsis detection, efficient hospital resource management, automated documentation of clinical encounters and decision support system (DSS). These use cases underscore the immense potential of AI/ML to enhance efficiency, accuracy, and outcomes across the healthcare spectrum (1). However, a very recent article raises essential considerations about the adoption and regulation of AI in clinical settings (2). Therefore, the integration of Artificial Intelligence (AI) in healthcare presents numerous opportunities and challenges. Conversely, there is a significant gap between clinician education regarding AI and the regulatory measures necessary for ethical deployment. To address this gap effectively, a structured, organized approach must be followed, encompassing clearly defined steps for both the education of clinicians and the establishment of rigorous regulatory frameworks. This paper argues that bridging this gap requires a dual approach: enhancing clinicians' understanding of AI technologies and treating AI systems as rigorously as pharmaceuticals through strict regulatory processes. By doing so, we can foster ethical and effective AI integration into clinical practice, ensuring patient safety and better healthcare outcomes. Here, we outline four key considerations that should guide the planning and regulation of AI integration in healthcare.

## 2 Expanding AI education among clinicians

Looking at the current clinical practice in healthcare according to the increase diffusion of AI is arguable the knowledge of physician about this new technology. In fact despite the increasing prevalence of AI in healthcare, many clinicians remain inadequately educated about what AI entails, its limitations and its implications too (1). Given that clinicians play a central role in patient care, a comprehensive AI education program should be a priority. Education initiatives should focus not only on how AI systems operate but also on how their regulatory framework should move on as for pharmaceuticals. This lack of understanding represents a significant barrier to the responsible adoption of AI technologies in clinical practice. To effectively bridge this gap, clinician education must cover both technical and ethical aspects of AI and should be part of medical degree course as well. A deeper understanding of the processes involved in validating AI tools can empower clinicians to participate meaningfully in discussions about the safety and efficacy of these systems. By building a foundational knowledge of AI, clinicians will be better prepared to evaluate and use AI tools within an ethical context and advocate for appropriate use and safety measures (3). This approach could be compared to that currently used to improve clinical or healthcare activity against antimicrobial resistance (AMR) or as for antimicrobial stewardship (4).

## 3 Understanding legal and ethical implications

Legal and ethical education must be extended beyond decision-makers and regulators to the clinicians who interact directly with AI systems. For clinicians to use AI responsibly, they must understand the legal implications, such as data privacy, accountability, and the ethical risks of biases^1^^,^^2^ (5). The recent European Union Artificial Intelligence Act lays out a regulatory framework for AI; however, this information is not often communicated to those on the front lines of healthcare (3). Educating clinicians about such regulatory initiatives is crucial to align their practical use of AI with ethical guidelines.^1^ As they navigate the clinical environment, an understanding of legal parameters will help them mitigate risks and ensure that AI integration prioritizes patient safety and respects privacy rights.

## 4 AI should be managed like a drug

There is a growing consensus that AI systems used in healthcare should be regulated in a manner similar to pharmaceuticals. Just as pharmaceuticals undergo a series of clinical trials to evaluate safety, efficacy, and ethical considerations, AI technologies should also follow rigorous validation processes before widespread implementation.

**Parallels to pharmaceutical validation**:

- **Phases of testing**: AI could adopt phases similar to drug development—preclinical (testing in controlled environments), Phase I (safety in small clinical settings), Phase II (efficacy trials), and Phase III (large-scale clinical testing). This structure would ensure that AI is tested for both safety and effectiveness in diverse, real-world environments.
- **Classification**: AI tools could be classified as drugs according to a system like ATC. For instance according to the type of AI tools type [drug discovery, precise analysis and interpretation of analysis, decision support system (DSS)] we could give them a code and a related regulatory activity.
- **Risk assessments**: Like drugs, AI must undergo thorough risk assessments, which include evaluating potential biases, unintended outcomes, and ethical implications.
- **Regulatory oversight**: A dedicated regulatory agency—potentially within the European Medicines Agency (EMA)—should combine expertise from both medical and engineering domains. This mixed approach would ensure a balanced evaluation of both the medical efficacy and technical performance of AI tools.

Finally, as in pharmaceuticals, AI systems should come with comprehensive documentation, including a “**Summary of Product Characteristics” and “Package Leaflet”** that outlines their intended use, limitations, and instructions for safe implementation. This approach will standardize AI information, enabling healthcare providers to make informed decisions based on clear guidance (6).

**Figure 1:**
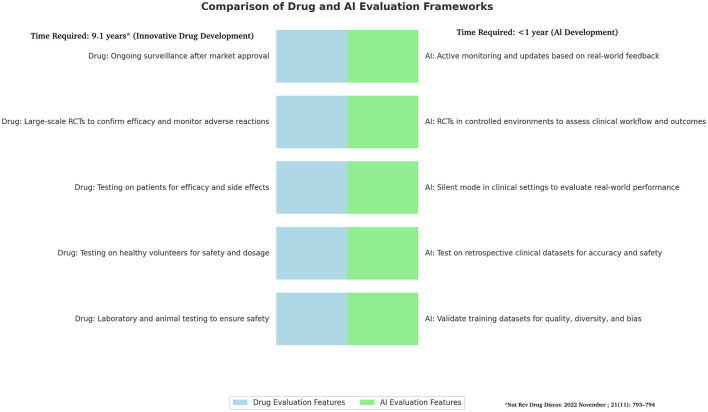
Comparison of drug and AI frameworks. Figure shows difference in frameworks of Drugs and AI development with several differences that highlight the need to find for AI quick as secure system for validation and deployment. The drug development timetable process is based on Brown et al. (7).

However, the two frameworks Pharmaceuticals and AI have some substantial differences (Table 1). In fact, while for innovative drug the time required to be approved tor clinical use is usually 9.1 years (7) AI technologies have shorter development cycles compared to traditional drug due to iterative model improvements and less dependency on long-term biological testing. This rapid pace requires an expedited but still rigorous framework for validation and deployment. Unlike drugs, AI tools benefit from iterative deployment, allowing for updates and enhancements after initial deployment based on real-world performance and user feedback, which means ongoing evaluation is critical. AI tools also offer significant advantages in scalability and adaptability. They can rapidly scale across diverse clinical environments and adjust to new datasets as they become available, setting them apart from more static pharmacological solutions. Therefore, AI should be managed and regulated as a Drug but according to a **Phase of Testing**, **Risk Assessment** and **Regulatory Oversight** being based to on specific tools for AI (8) (Figure 1).

**Table 1:** Key differences between drug and AI evaluation.

**Aspect**	**Drugs**	**AI systems**
Development	Fixed chemical or biological entity, formula remains constant throughout.	Iterative, data-driven models that evolve with new data and retraining.
Testing approach	Static population and fixed protocols in controlled trials.	Dynamic testing, adapting to real-world scenarios and diverse populations.
Deployment	Single approval process with fixed guidelines for use.	Continuous deployment with ongoing validation, monitoring, and updates.
Regulation	Linear, phase-based evaluation (preclinical to post-marketing).	Cyclical, iterative evaluation requiring periodic reassessments and real-time monitoring.
Impact	Direct physiological or biochemical impact on patients.	Indirect influence through decision-making, workflow optimization, and recommendations.
Failure impact	Specific adverse effects, often localized to the drug.	Systematic risks like bias amplification, incorrect predictions, or workflow disruption.
Lifecycle	Fixed lifecycle, with few post-market changes.	Continuous lifecycle, requiring retraining, updates, and adaptation to new contexts.
Validation metrics	Efficacy and safety measured against standard endpoints in trials.	Performance measured by accuracy, precision, recall, and real-world outcome improvements.
Monitoring	Post-marketing surveillance for adverse effects.	Continuous monitoring with feedback loops and performance optimization.
Human involvement	Limited after initial trials (patient compliance is key).	High, with human-in-the-loop design during development, deployment, and monitoring.

Figure shows difference in frameworks of Drugs and AI development with several differences that highlight the need to find for AI quick as secure system for validation and deployment.

## 5 Economic factors should not compromise ethical standards

While the commercialization of AI in healthcare is inevitable, ethical standards must not be compromised for economic gain. Proper governance, including stringent oversight by a mixed medical-engineering regulatory body, will ensure that AI systems adhere to healthcare's core ethical principles. Regulatory frameworks must ensure that **economic motivations should drive innovation but must always be secondary to patient safety**, quality of care, and ethical obligations. This balance will foster trust in AI technologies among both clinicians and patients (9).

## 6 Conclusion

Artificial intelligence (AI) offers immense potential to transform healthcare, from improving patient outcomes to enhancing clinical workflows and driving medical innovation. However, its successful integration into healthcare requires overcoming several key challenges, including gaps in clinician education, the need for ethical governance, and the absence of tailored regulatory frameworks. This paper argues for regulating AI with the same rigor as pharmaceuticals, incorporating validation phases, risk assessments, and detailed documentation, while adapting these processes to AI's fast and iterative development cycles.

A critical first step is educating clinicians about AI. Many healthcare professionals lack a clear understanding of how AI systems work, their limitations, and the ethical and legal issues they raise. Comprehensive training programs are needed to build this knowledge. Such education should focus not only on technical aspects but also on teaching clinicians how to assess AI tools for safety, effectiveness, and ethical implications. This would empower clinicians to confidently use AI in their practice, ensuring that its benefits are fully realized while safeguarding patient trust.

Equally important is the development of regulatory frameworks that match AI's unique characteristics. Unlike pharmaceuticals, which follow a linear path from development to deployment, AI evolves continuously. Regulations must therefore balance rapid innovation with robust oversight, ensuring AI systems are safe, effective, and free from bias. This approach requires collaboration between healthcare professionals, technology developers, and regulators to create guidelines that are both practical and ethical.

Economic pressures should not overshadow the ethical responsibilities involved in AI integration. While commercialization drives innovation, patient safety and quality of care must always come first. By fostering partnerships between industry and healthcare that prioritize ethical principles, we can build trust in AI technologies and their use in clinical practice.

To address these challenges, the following steps should guide AI integration:

1. Establish clear and practical processes for validating and monitoring AI systems, drawing inspiration from pharmaceutical regulation but tailoring these to AI's specific needs.
2. Develop accessible training programs for clinicians, focusing on building their confidence and competence in using AI tools.
3. Support pilot projects and real-world case studies to demonstrate how AI can be safely and effectively used in different healthcare settings.

By taking this structured and adaptive approach, we can bridge the current gaps in AI integration. This will ensure that AI evolves responsibly, supporting healthcare providers and benefiting patients while maintaining the highest ethical standards. Ultimately, this balanced strategy will enable AI to fulfill its promise of transforming healthcare in a way that is safe, effective, and equitable (10, 11).

## Footnotes

^1^ https://eur-lex.europa.eu/eli/reg/2017/746/oj

^2^ https://www.isaca.org/resources/isaca-journal/issues/2023/volume-2/the-potential-impact-of-the-european-commissions-proposed-ai-act-on-smes
